# Author Correction: The chicken chorioallantoic membrane as a low-cost, high-throughput model for cancer imaging

**DOI:** 10.1038/s44303-025-00118-7

**Published:** 2025-10-18

**Authors:** Lydia M. Smith, Hannah E. Greenwood, Will E. Tyrrell, Richard S. Edwards, Vittorio de Santis, Friedrich Baark, George Firth, Muhammet Tanc, Samantha Y. A. Terry, Anne Herrmann, Richard Southworth, Timothy H. Witney

**Affiliations:** 1 School of Biomedical Engineering & Imaging Sciences, King’s College London, London, UK; 2 Institute of Systems, Molecular and Integrative Biology, University of Liverpool, Liverpool, UK

**Keywords:** Cancer imaging, Positron-emission tomography

Correction to: *npj Imaging* 10.1038/s44303-023-00001-3, published online 29 November 2023

In this article, the competing interest statement was incorrectly given as ‘The authors declare no competing interests’ but should have been ‘Author T.H.W. is Editor-in-Chief of npj Imaging. The remaining authors have no competing Interests. The original article has been corrected.’

