# Supplementary material for: The chicken chorioallantoic membrane as a low-cost, high-throughput model for cancer imaging
Source: Npj Imaging. Author manuscript; Available in PMC 2024 Jan 18. (PMC7615542; doi:10.1038/s44303-023-00001-3)
Supplement: Supplementary data [file EMS192709-supplement-Supplementary_data.pdf]

Supplemental data: The Chicken Chorioallantoic Membrane as a Low-Cost, High-Throughput Model for Cancer Imaging

Lydia M. Smith, Hannah E. Greenwood, Will E. Tyrrell, Richard S. Edwards, Vittorio de Santis, Friedrich Baark, George Firth, Muhammet Tanc, Samantha Y.A. Terry, Anne Herrmann, Richard Southworth & Timothy H. Witney

SUPPLEMENTAL FIGURES AND LEGENDS

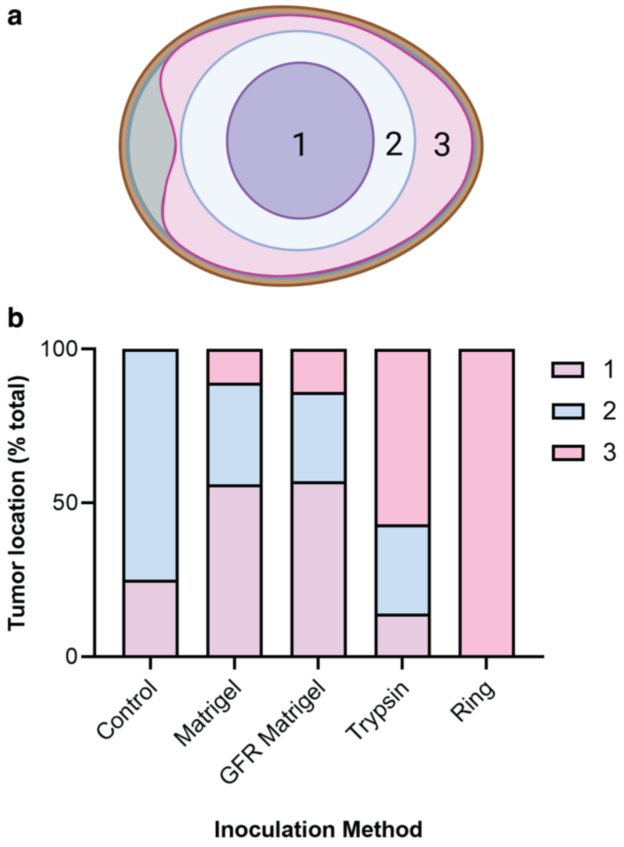

SUPPLEMENTAL FIGURE 1. Location of chick CAM tumor growth with various inoculation methods. a. Schematic illustrating the possible positions of tumor growth within the egg. Location 1: the center of the CAM. Location 2: the edges of the CAM. Location 3: the inside of the shell. b. Location of grown tumors based on inoculation method and matrix.

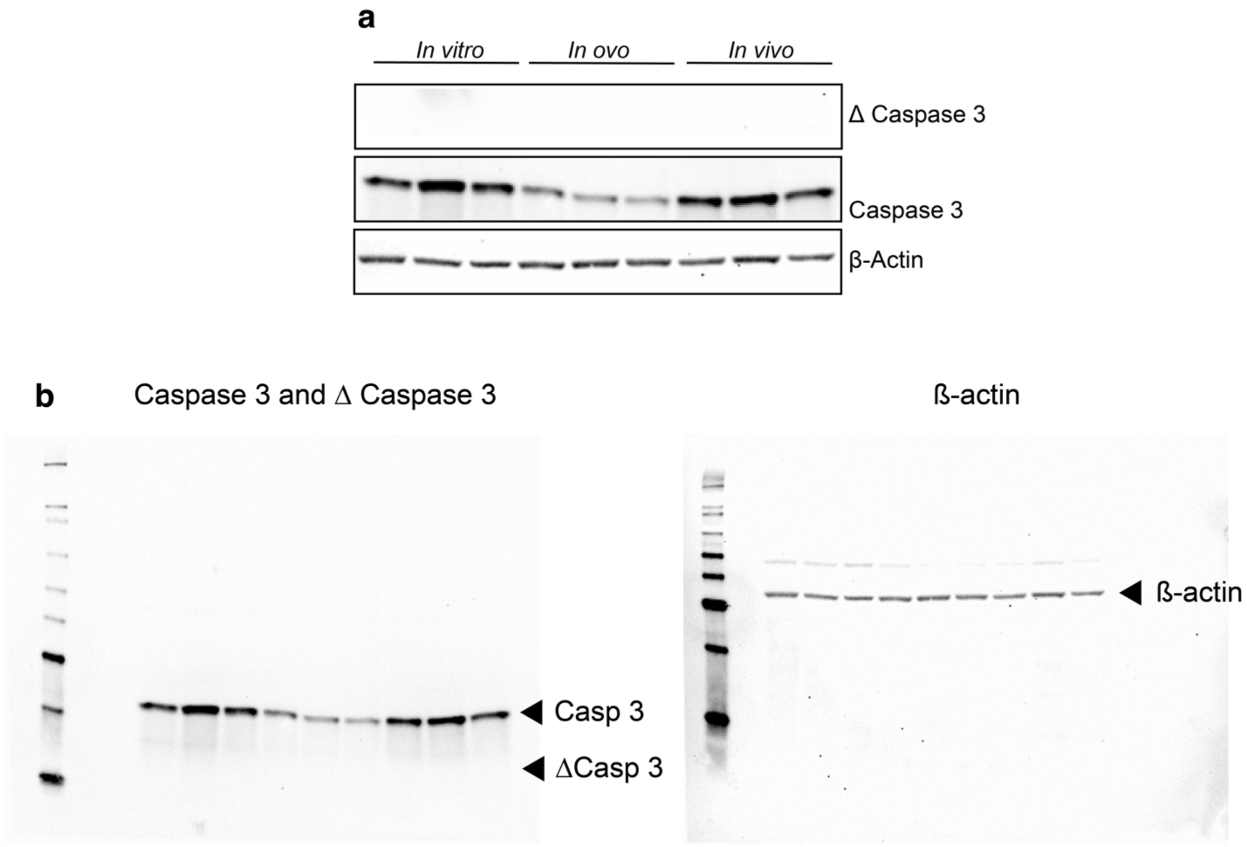

SUPPLEMENTAL FIGURE 2. Tumors grown *in ovo* have low baseline levels of apoptosis. a. Cropped western blots illustrating cleaved caspase 3 levels (Δ) in NCI-H460 Fluc cells grown *in vitro*, *in ovo*, and *in vivo*. Actin and total caspase 3 were used as controls. b. Corresponding uncropped original blots.

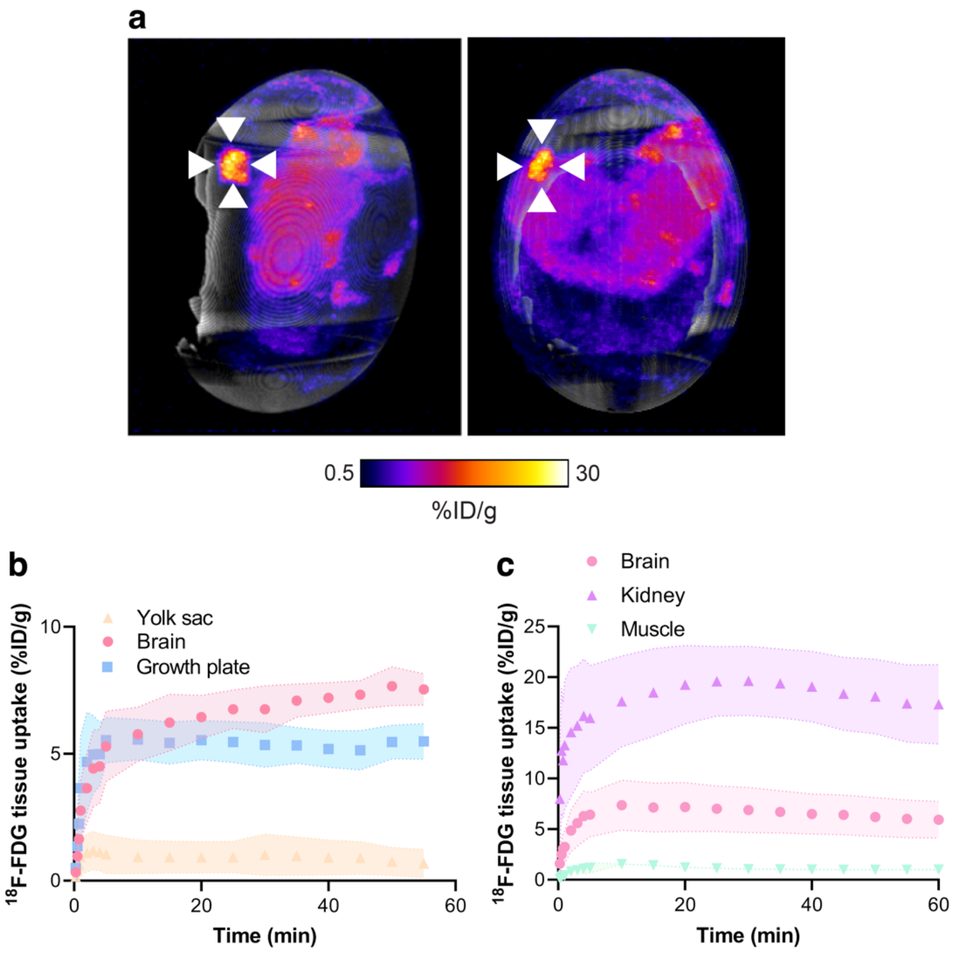

SUPPLEMENTAL FIGURE 3. ^{18}F-FDG PET/CT maximum intensity projections and healthy tissue time activity curves. a. ^{18}F-FDG PET/CT maximum intensity projection (0 – 60 min p.i.) from a chick CAM with an NCI-H460 Fluc xenograft tumor (white arrow heads). b. TAC for ^{18}F-FDG tissue uptake from the organs of the embryo. n = 7. c. TAC for ^{18}F-FDG tissue uptake in mice. n = 9. Shaded regions represent one standard deviation.

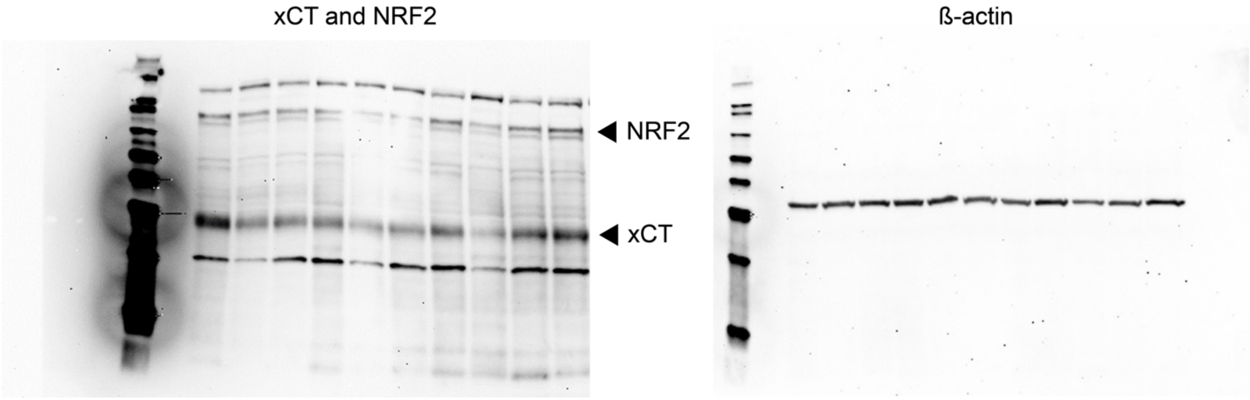

SUPPLEMENTAL FIGURE 4. Full, uncropped western blots for xCT, NRF2, and actin from Figure 4d.

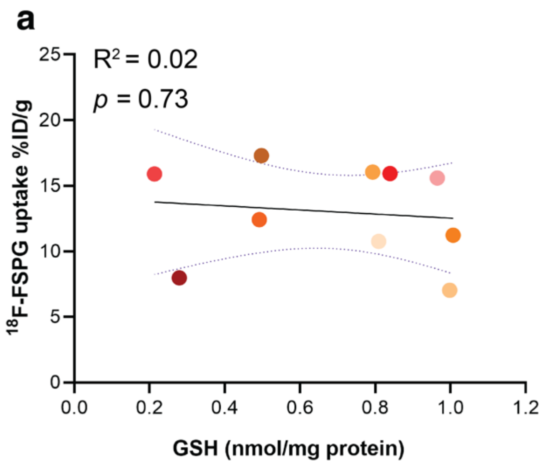

SUPPLEMENTAL FIGURE 5. ^{18}F-FSPG retention in the chick CAM doesn't correlate with tumor size or GSH concentration. a. Correlation plot of tumor ^{18}F-FSPG retention *vs.* intracellular GSH. b. Correlation plot of tumor size *vs.* tumor ^{18}F-FSPG retention.

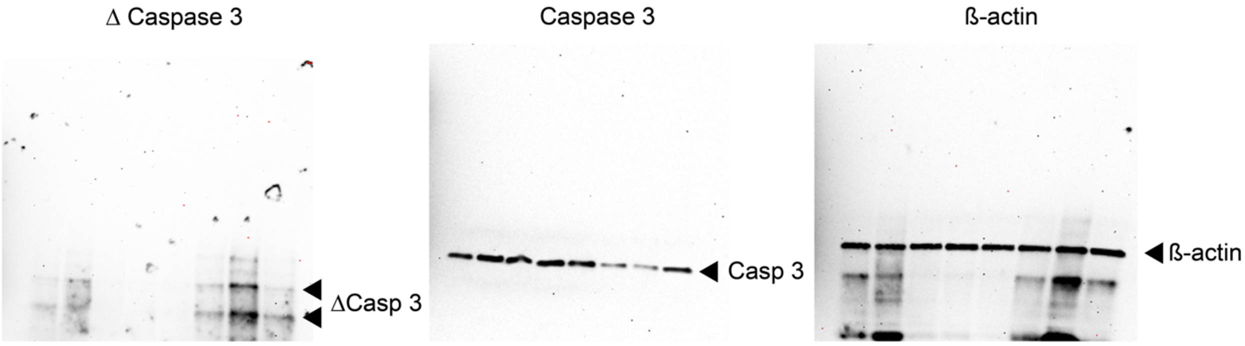

SUPPLEMENTAL FIGURE 6. Full, uncropped western blots for cleaved caspase 3 (Δ), caspase 3, and actin from Figure 7c.

REFERENCES

1. Greenwood HE, Edwards R, Koglin N, et al. Radiotracer stereochemistry affects substrate affinity and kinetics for improved imaging of system xC-in tumors. *Theranostics.* 2022;12:1921.
